# Kinetics of Structural Transitions Induced by Sodium Dodecyl Sulfate in α-Chymotrypsin

**DOI:** 10.1021/acsomega.3c07256

**Published:** 2023-12-13

**Authors:** Karolina Stachurska, Urszula Marcisz, Maciej Długosz, Jan M. Antosiewicz

**Affiliations:** Biophysics Division, Institute of Experimental Physics, Faculty of Physics, University of Warsaw, Pasteura 5 Street, 02-093 Warsaw, Poland

## Abstract

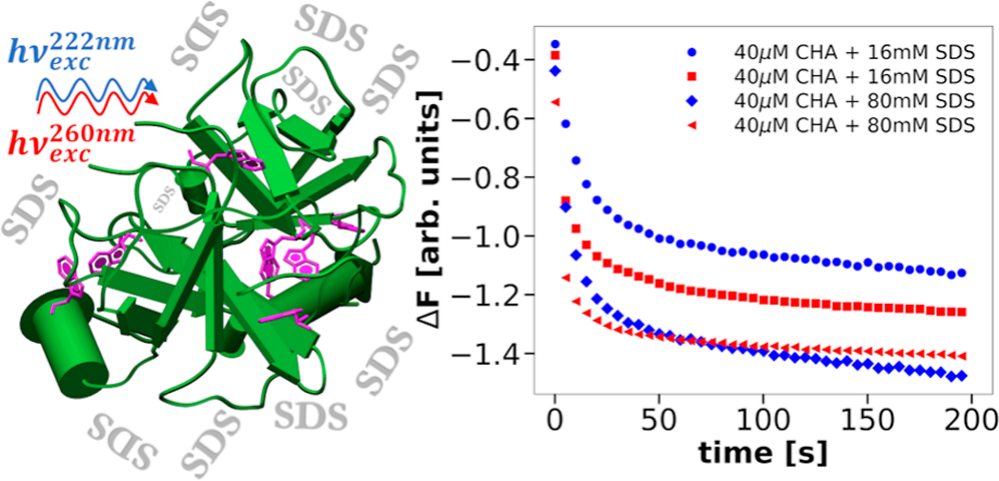

The temporal changes in circular dichroism at 222 and 260 nm were recorded by using stopped-flow spectroscopy after mixing α-chymotrypsin solutions with sodium dodecyl sulfate solutions. Simultaneously with the circular dichroism signal, the fluorescence emission was recorded. Changes in the secondary and tertiary structures of chymotrypsin induced by sodium dodecyl sulfate are characterized by either three or four one-way reactions with relaxation amplitudes and times precisely determined by an advanced numerical procedure of Kuzmič. Quantitatively, transitions within the secondary and tertiary structures of the protein are significantly different. Moreover, changes in the tertiary structure depend on the type of recorded signal (either circular dichroism or fluorescence) and the wavelength of the incident radiation. The latter observation is particularly interesting as it indicates that the contributions of protein’s different tryptophans to the total recorded fluorescence depend on the excitation wavelength. We present several results justifying this hypothesis.

## 1 Introduction

Investigations of protein–surfactant interactions have a long history owing to their widespread application in the pharmaceutical, food, and cosmetic industries and their importance in studies in the field of basic physical chemistry of proteins.^1−7^ Sodium dodecyl sulfate (SDS) has been most frequently adopted as a representative surfactant.^2,8−10^ This anionic detergent is known to unfold globular proteins. While the majority of studies on protein–SDS systems focus on their equilibrium properties, kinetic aspects of SDS–protein interactions have become a more common subject.

Kinetic investigations of the effects of SDS on the structure and dynamics of proteins started in the early 80s, when Takeda and co-workers^11−14^ used stopped-flow spectrometry with circular dichroism (CD) and absorbance signals and the pressure-jump method with conductivity measurements to study structural changes induced in proteins by SDS. Progress curves recorded in experiments for proteins like α-chymotrypsin,^11^ δ-chymotrypsin,^12^ and bovine serum albumin^14^ were classified as monoexponential. The observed rate constant, *k*_obs_, was initially increasing steeply with the SDS concentration and then stabilizing at a plateau at some higher SDS concentration. The 2015 review article by Takeda and Moriyama^9^ summarizes the main results of these early studies. As they emphasized, the original purpose of undertaking this research was to compare the rate of changes in the secondary structure of the protein with the rate of changes in the tertiary structure that occur under the influence of added SDS. They found^11,12,14^ that the secondary structure and the tertiary structure of proteins change simultaneously, the time scales of these changes are similar, and they occur in one irreversible step.

Moreover, Takeda and co-workers noticed, in capillary electrophoresis (CE) experiments, discontinuous mobility changes of surfactant–protein complexes. These were first reported by Sasa and Takeda,^15^ who described conformational changes of bovine serum albumin in the presence of SDS and revealed four distinct mobilities at different SDS concentrations related to distinct protein–SDS complexes. According to Takeda and Moriyama,^9^ the discontinuous mobility changes of surfactant–protein complexes observed in CE experiments with an increasing SDS concentration indicate that only the complexes of a given protein with some specific numbers of SDS ions are present in the solution. In other words, when the surfactant concentration is insufficient to saturate the binding, the binding occurs equally to every protein, and the binding amount of surfactant ions equally increases on each protein with an increase in surfactant concentration.

Kinetic investigations of structural transitions in proteins caused by SDS or other surfactants have also been undertaken by other research groups.^8,16−32^

Particularly impressive are recent investigations by the Otzen and Pedersen groups,^29−31^ who concluded their studies by presenting a detailed molecular picture of the changes occurring in protein molecules after SDS binding, including the analysis of the structures formed by the surfactant.^33^ They used CD and fluorescence to compare the kinetics of changes in the secondary and tertiary structures due to the addition of SDS, and additionally, they followed changes in the overall architecture of the protein–surfactant complexes by synchrotron small-angle X-ray scattering (SAXS). By combining stopped-flow mixing of protein and surfactant solutions with monitoring synchrotron SAXS, CD, and fluorescence, the Otzen and Pedersen groups documented two-step (at least) unfolding kinetics of α-lactalbumin^29^ and β-lactoglobulin.^30^ For the S6 protein,^31^ the Otzen group showed multiple-step unfolding relaxation using stopped-flow fluorescence spectroscopy.

In earlier studies, Otzen and co-workers^18,19,23^ interpreted the dependence of the observed conformational transition rate constant on SDS concentration based on the following minimal mechanism, which involves rapid binding completed within the dead time of stopped-flow mixing and the subsequent global unfolding reaction (eq 1; we slightly changed the original equation so that it would be consistent with the equations shown in the present work):
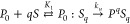
1

*P*_0_ is the native protein, *S* is a surfactant ion, *q* is the number of SDS molecules bound within the dead time of the stopped-flow apparatus, *P*_0_:*S*_*q*_ is the protein–surfactant complex from which unfolding occurs, *K*_1_ is an apparent association constant ([*P*_0_:*S*_*q*_]/[*P*_0_][*S*]^*q*^), and *k*_*u*_ is the rate constant for unfolding. The one-step SDS binding in reaction eq 1 has a symbolic meaning. The binding of surfactants to proteins consists of successive bimolecular steps that equilibrate on a short time scale. These fast binding steps are followed by an irreversible one-step structural change of the complex. We can therefore assume that mixing a micromolar protein solution with an SDS solution at a concentration of 10–100 mM acts as a trigger, initiating unidirectional structural transformations of the protein.

Here, we investigate the kinetics of SDS-induced transitions in the tertiary and secondary structure of α-chymotrypsin (CHA), one of the proteins studied by the Takeda group,^11^ using stopped-flow spectrometry with simultaneous recording of CD and fluorescence signals, which guarantees that the phenomena are observed under exactly the same conditions and at exactly the same time. Such simultaneous measurements in kinetic experiments have already been described by other researchers.^34,35^ Takeda^12^ made his CD and absorbance measurements in separate experiments, but the solvents used, concentrations of chymotrypsin and SDS, and temperature in both experiments were the same. Otzen and colleagues also studied the kinetics of secondary and tertiary structure transitions in separate experiments.

The time scale of SDS-induced structural transitions of α-chymotrypsin is sufficiently slow so that stopped-flow progress curves are characterized by a high signal-to-noise ratio. As far as we were able to gather from the published literature, stopped-flow investigations of the unfolding of α-chymotrypsin^11^ and δ-chymotrypsin^12^ are the only kinetic studies on SDS-induced structural changes in chymotrypsin. In comparison to those studies, our experimental data are of higher quality. Moreover, we analyze recorded CD and fluorescence progress curves using a reliable and well-established tool, the DynaFit 4 program of Kuzmič,^36,37^ which numerically integrates differential equations describing any given reaction equation, optimizes rate constants, and, based on sophisticated methods for statistical model discrimination, finds the reaction equation that best fits the experimental data. We compare the kinetics of secondary and tertiary structure transitions of α-chymotrypsin and discriminate between different unfolding models involving multiple steps, rates, and amplitudes.

We conclude that the kinetics of changes in the secondary structure of chymotrypsin differ substantially from the kinetics of changes in its tertiary structure. In addition, the values of amplitudes and rates characterizing the multistep tertiary structure transitions depend on the observation method (either CD or fluorescence signal). Even more notably, values of rates and amplitudes, derived from fluorescence progress curves, depend on the excitation wavelength (222 vs 260 nm). We thus formulate a novel hypothesis that different fluorescent chromophores in a protein make different contributions to the total measured protein fluorescence at different excitation wavelengths. We present several results that prove the validity of this hypothesis, but its full verification is a challenge that requires separate research.

## 2 Materials and Methods

All chemicals used in the present work were obtained from ROTH. Chymotrypsin (ROTH, *ge*1 000 USP-U/mg, for biochemistry, Art-Nr. 0238.3), sodium dodecyl sulfate (SDS, ROTH, ≥99%, for electrophoresis, for biochemistry and molecular biology, Art-Nr. 2326.1), sodium dihydrogen phosphate monohydrate (ROTH, *ge*98%, p.a., ACS, Art.-Nr. K300.1), disodium hydrogen phosphate dehydrate (ROTH, ≥ 98%, p.a., ACS, Art.-Nr. 4984.2), sodium chloride (ROTH, ≥ 98%, p.a., ACS, ISO, Art.-Nr. 3957.1), and l-tryptophan (ROTH, ≥ 98.5%, Ph. Eur., for biochemistry, Art.-Nr. 4858.1).

Chymotrypsin, SDS, and l-tryptophan were dissolved in a sodium phosphate buffer (20 mM, pH 6.4 with 5 mM NaCl), prepared using ultrapure Millipore Milli-Q water (resistivity 18.2 MΩ·cm). All final solutions used in experiments were prepared by diluting appropriate stock solutions of chymotrypsin, SDS, and l-tryptophan. The concentration of the stock protein solution was 44.444 μM, controlled spectrophotometrically using ϵ_282 nm_ = 51,000 M^–1^ cm^–1^. Such a concentration of the chymotrypsin stock solution was dictated by the need to have *parent* 40 μM protein solutions, either without SDS or with the desired amount of SDS, that would be obtained by diluting the same stock solution, for further mixing experiments in the stopped-flow spectrometer. The concentration of the stock SDS solution was 320 mM. Using this solution, SDS *parent* solutions (16, 32, 48, 64, and 80 mM) were prepared. This gives a range of final SDS concentrations when mixed with the chymotrypsin solution from 8 to 40 mM, which is a range similar to that used in Takeda’s work on α- and δ-chymotrypsin.^11,12^l-tryptophan concentration in the stock solution was 40 μM and was determined spectrophotometrically using ϵ_280 nm_ = 5600 M^–1^ cm^–1^. Prior to stopped-flow experiments, all samples were vacuum degassed for 60 min.

While we could not find relevant data in the literature, based on the report by Fuguet et al.,^38^ it is safe to assume that in the 20 mM phosphate buffer with the addition of 5 mM NaCl, pH 6.4, and temperature of 20 °C, the critical micelle concentration (cmc) of SDS is no more than 3 mM. Thus, in all our experiments, the SDS concentration is substantially above the cmc.

### 2.1 Spectrophotometric Measurements

UV–vis absorption spectra were recorded by using the UV-2401-PC Shimadzu spectrometer. The fluorescence emission spectra were recorded with a Cary Eclipse Fluorescence Spectrophotometer (Agilent Technologies) in a quartz 10 × 10 mm cuvette. The excitation wavelength was set to either 222 or 260 nm. Spectra were recorded from 300 to 550 nm. To ensure measurement conditions corresponding to the recording of fluorescence in our kinetic studies (see below), the fluorescence spectra were collected through a 320 nm cutoff filter (Schott WG 320). The widths of the emission and excitation slits were set to 5 nm, the detector voltage was 600 V, and the scanning speed was 30 nm/min. The cell was thermostated with a Cary Single Cell Peltier Accessory (Agilent Technologies). One emission spectrum was recorded for each sample.

CD spectra were collected using a Chirascan Plus (Applied Photophysics Ltd.) spectrometer. This spectrometer enables simultaneous measurement of the CD, absorbance, and fluorescence excitation spectra. For the far-UV range (200–250 nm), a 1 mm cell was used. For the near-UV range (250–345 nm), a 10 mm cell was used. The CD spectra of α-chymotrypsin solutions or the corresponding solvent were scanned with 0.5 s integration, 0.5 nm step resolution, and 1 nm bandwidth. Six scans were performed and averaged. Before spectra measurements, the CD baseline was recorded with an empty cell holder and with a 3 s integration. From each recorded spectrum of α-chymotrypsin solution, the corresponding smoothed buffer spectrum was subtracted. Buffer spectra were smoothed by the Savitsky-Golay^39^ method with a window size of 11 using the Pro-Data Chirascan 4.1 (Applied Photophysics Ltd.) software.

All spectroscopic measurements were carried out at 20 °C.

### 2.2 Kinetic Experiments

For the kinetic experiments, we used the SF.3 stopped-flow accessory for the Chirascan CD spectrometer, which enables the simultaneous collection of CD, absorbance, and fluorescence signals after mixing two selected solutions. We also performed additional kinetic experiments with fluorescence measurement using an SX20 spectrofluorimeter from Applied Photophysics Ltd.

In essential experiments using the SF.3 stopped-flow accessory, the mixed solutions were excited by the light of either 222 (far-UV experiments) or 260 nm (near-UV experiments) wavelength, with a 7 nm bandwidth. The reference CD signal was measured with the stopped-flow mixing cell filled with buffer by using the baseline command.

For the far-UV CD stopped-flow experiments, the optical path length was 2 mm, and for the near-UV CD stopped-flow experiments, the optical path length was 10 mm. The emission was simultaneously collected at 90° to the excitation beam using a 320 nm cutoff filter (Schott WG 320). The emission pathway was 1 mm for both wavelengths. The voltage of the fluorescence photomultiplier tube, 350 V, resulted in an output signal of 8 V after mixing 40 μM α-chymotrypsin solution with the phosphate buffer. The photomultiplier tube voltage of 350 V was kept unchanged over the whole range of our experiments.

We performed two series of stopped-flow experiments. In the first series, we mixed a 40 μM α-chymotrypsin solution either with the buffer or with 16, 32, 48, 64, or 80 mM SDS diluted in the same buffer. In the second series, we mixed a solution of 40 μM α-chymotrypsin containing the addition of *x* mM SDS and another solution of SDS having a concentration of (80 – *x*) mM.

For each pair of mixed solutions, we averaged 12 reaction progress curves recorded in the stopped-flow experiments for the 222 nm excitation wavelength and 25 reaction progress curves recorded for the 260 nm excitation wavelength. A higher number of individual progress curves in the latter case was required because the addition of SDS resulted in a smaller change in the recorded signal. All solutions were prepared in a degassed buffer. All preparations and measurements were carried out at a controlled temperature of 20 °C.

### 2.3 Analysis of Progress Curves

The influx of solutions into the mixing chamber of the stopped-flow spectrometer triggers structural changes in the protein. The binding process ends within the spectrometer dead time, and the (substantial) number of surfactant molecules bound to the protein is not known. Therefore, the description of the reactions taking place in the mixing chamber does not include the binding step.

We discriminate between unimolecular reaction equations differing in the number of steps, *n*
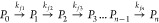
2

We note that it is not possible to distinguish between irreversible processes shown in reaction eq 2 and corresponding reversible processes shown in reaction eq 3
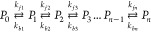
3based solely on the progress curves.

From a mathematical standpoint, reactions eqs 2 and 3 lead to systems of differential equations that can be solved analytically (assuming appropriate initial conditions). In both cases, the solution has the form of a multiexponential function. In the case of the irreversible reaction, each exponential decay constant corresponds to the rate of a given unimolecular step (*k*_f*i*_), whereas in the case of the reversible reaction, exponential decay constants are some functions of forward and back rates (*k*_f*i*_, *k*_b*i*_). For example, for a two-state unimolecular process, either reversible or irreversible, , the resulting monoexponential progress curve is characterized by the so-called observed rate constant, defined either as *k*_obs_ = *k*_+1_ + *k*_–1_ for the reversible reaction or *k*_obs_ = *k*_+1_ for the irreversible reaction. It is not possible to discriminate between the two cases without an additional input, such as the value of the equilibrium constant *K* = *k*_+1_/*k*_–1_ or the contributions of species A and B to the signal measured in kinetic experiments. Distinguishing between reversible and irreversible unimolecular transitions is even more difficult for processes involving multiple steps, as knowledge of all equilibrium constants may not be sufficient to detect reversibility based on fitting corresponding progress curves.

The recorded progress curves were analyzed using the DynaFit 4 program of Kuzmič.^36,37^ Functions resulting from the numerical integration of the differential equation (with optimal values of rate constants) corresponding to a given reaction mechanism as well as multiexponential curves were fitted to experimental traces. In some of the analyzed cases (see below), a linear drift was included in the fitting to accommodate the influence of tryptophan photobleaching on the registered signal.^23,40^

For the selection of the most probable reaction mechanism, we used the model discrimination algorithm implemented in DynaFit. This algorithm is based on the calculation of the second-order Akaike’s information criterion^41^ and the Bayesian information criterion^42,43^ parameters for all considered models. Selection among the proposed models was additionally based on consideration of empirical confidence intervals at a 1% increase in the residual sum of squares.^44^

Apart from performing fits, the DynaFit program was also used to simulate reaction progress curves for particular models of SDS-induced CHA conformational transitions.

## 3 Results and Discussion

Based on the available literature, we consider it physically sound to assume that all structural transitions of α-chymotrypsin observed in our stopped-flow experiments are unimolecular processes, either irreversible or reversible, triggered by fast (completed within the dead time of the apparatus) binding of SDS. Therefore, each progress curve obtained after mixing solutions of the protein and SDS must be fitted separately. This is a qualitatively different situation than that encountered, for example, in the case of binding ligands by receptors possessing a small number of binding sites. In such a case, by choosing appropriate concentrations, the binding process can be made sufficiently slow, and as a consequence, one can fit simultaneously several progress curves obtained for different initial concentrations of the reagents. Here, structural transitions of α-chymotrypsin start to be visible only if the initial concentration of SDS exceeds about 100 μM, and even larger concentrations of SDS are required to observe changes in CD spectra.

### 3.1 Stationary CD and Fluorescence Spectra

Figure S1 presents the far- and near-UV CD spectra of 20 μM solutions of α-chymotrypsin and their changes in the presence of 8–40 mM SDS. All the spectra qualitatively agree with those presented in the literature.^45−47^ Substantial changes resulting from the addition of SDS, visible around 222 and 260 nm, were employed in our stopped-flow mixing experiments with the registration of the CD signal. The SDS-induced change in the CD spectra visible around 208 nm was not suitable for stopped-flow experiments because at this wavelength, the absorption of the solution in the stopped-flow apparatus mixing cell is too large.

The change observed in the far-UV region indicates that SDS increases the population of α-helices in CHA. On the other hand, changes observed in the near-UV point to some transitions in the tertiary structure of α-chymotrypsin upon the addition of SDS, and so do changes in the fluorescence spectra.

Figure S2 shows the stationary fluorescence spectra, recorded with a 320 nm cutoff filter, for excitation wavelengths of 222 and 260 nm for 20 μM solutions of CHA in phosphate buffer with and without an addition of 40 mM SDS. Much lower fluorescence intensity results from the 222 nm excitation wavelength than from 260 nm, which is mainly due to the much greater inner-filter effect in the first case. According to a previous study,^48^ the measured intensity, *I*_measured_, should be corrected for the effects due to the absorption of the fluorescent molecule at the excitation (exc) and emission (em) wavelengths in the following fashion
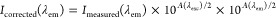
4where *A*(λ) is the absorption of radiation with wavelength λ. Due to the fact that proteins do not absorb the radiation of their fluorescence, this correction factor is the same for each wavelength of the emitted fluorescence light; therefore, there is no influence of the internal filter effect on the shape of the fluorescence band. As a consequence, one should be able to superimpose normalized fluorescence spectra recorded at different concentrations.

### 3.2 Progress Curves Recorded in Kinetic Experiments

In our kinetic experiments conducted with the SF.3 stopped-flow accessory for the Chirascan CD spectrometer, we mixed 40 μM solutions of CHA with 16–80 mM SDS solutions. For each pair of mixed solutions, the CD and fluorescence signals were recorded simultaneously. The same CHA concentrations were used for both considered excitation wavelengths so that the kinetics of protein conformational changes could be compared in a physically meaningful way by analyzing the recorded reaction progress curves.

The CD progress curves recorded in the far-UV range give us insight into the kinetics of secondary structure transformations. However, the analysis of the CD signal in the near-UV and the fluorescence signal, regardless of the wavelength of the incident radiation, allows us to determine the kinetics of the tertiary structure transformations.

As might be expected, SDS in phosphate buffer undergoes a structural transition as its concentration increases from 8 to 40 mM, from spherical micelles to rod micelles.^49^ This may affect the molecular environment of α-chymotrypsin and its interactions. For this reason, we do not discuss below the changes in the recorded reaction progress curves as the SDS concentration increases. However, we compare reaction progress curves recorded with different spectroscopy for the same pairs of chymotrypsin and SDS concentrations mixed in a stopped-flow spectrometer mixing cell.

Insight into the structural details of chymotrypsin and SDS complexes could be obtained from molecular modeling, for example, molecular dynamics methods, but in our experiments, we do not obtain any results that could be used to verify the reliability of the simulation results. In our approach, adding SDS to the chymotrypsin solution serves as a trigger to initiate structural changes in the protein.

The results of the kinetic studies are presented below in the three sections. First, we compare CD reaction progress curves recorded at the 222 and 260 nm excitation wavelengths. Then, we compare the fluorescence reaction progress curves recorded at these two lengths of the incident light. Finally, we compare CD and fluorescence reaction progress curves recorded at a 260 nm excitation wavelength.

#### 3.2.1 Comparison of Kinetics of Secondary and Tertiary Structure Changes. CD Measurements at 222 and 260 nm Excitation Wavelengths

We expect that the CD reaction progress curves obtained after mixing CHA solutions with the buffer will not show any dependence on time. However, as evidenced in Figure S3, slight disturbances may occur in the registered signal during the initial recording phase. For this reason, from the progress curves obtained after mixing the protein and SDS solutions, we subtract the curve obtained after mixing the protein with the buffer. Relative signals obtained in this fashion represent structural transitions in proteins induced by SDS.

Figure S4 shows all CD relative progress curves in the far- and near-UV ranges. As can be seen, increasing SDS concentration increases the overall rate of processes responsible for the observed CD signals and also increases the relaxation amplitude. For the 260 nm excitation wavelength, time courses measured for different SDS concentrations saturate at almost the same value. Moreover, recorded progress curves (with the exception of the one obtained for the lowest SDS concentration) overlap. In the case of the 222 nm excitation wavelength, saturation is not observed, and the recorded progress curves are clearly distinguishable. As the CD signal in the far-UV reflects the helical content in the CHA, we may conclude that the helical fraction increases and reaches a limiting value for the highest concentration of SDS considered.

**Figure 1:**
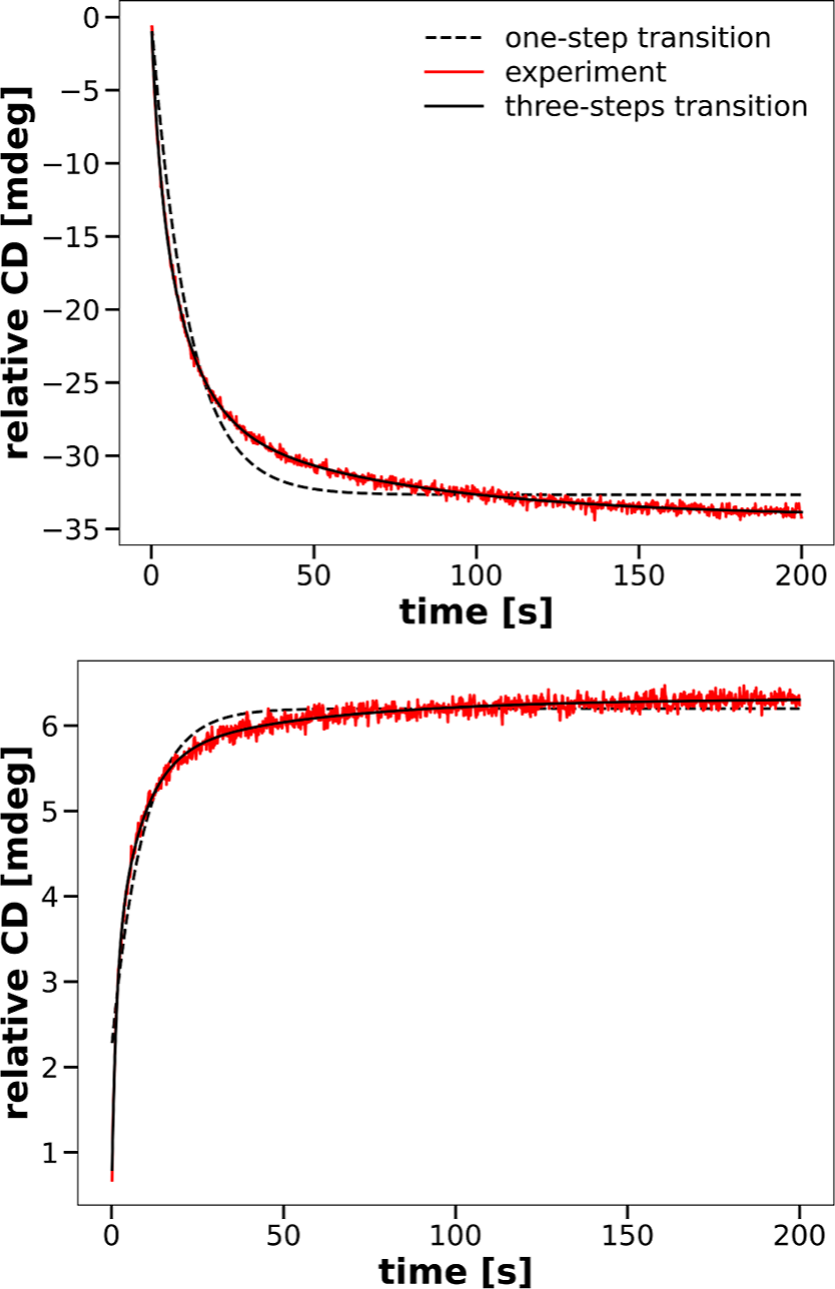
Fits of relative CD progress curves obtained after mixing the 40 μM solution of α-chymotrypsin with a 64 mM solution of SDS (top: 222 nm excitation wavelength; bottom: 260 nm excitation wavelength).

Figure 1 presents exemplary fits obtained with the DynaFit 4 program of the CD progress curves recorded after mixing the 40 μM α-chymotrypsin solution with the 64 mM solution of SDS for 222 and 260 nm excitation wavelengths. Fits correspond to an irreversible three-step process. The discrimination procedure implemented in the DynaFit 4 program indicated that such reaction mechanisms are the most probable. This also holds true for the remaining concentrations of SDS used in our experiments; three steps are required and sufficient to fit all of the CD progress curves.

For comparison sake, the best fits of one-step transition models are also shown in Figure 1. It is obvious that SDS-induced structural transitions of CHA must involve more than one step.

**Figure 2:**
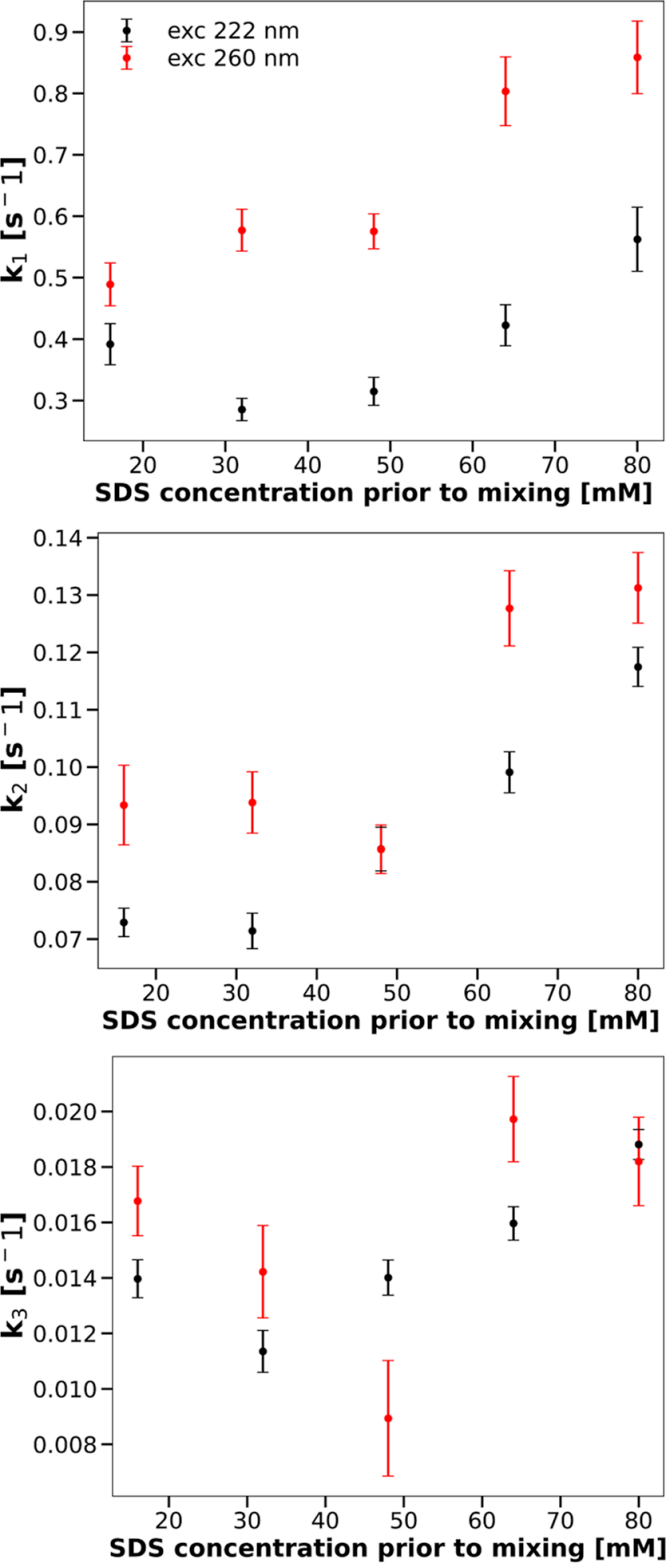
Rate constants of the transitions in the secondary (black symbols, excitation wavelength of 222 nm) and the tertiary (red symbols, excitation wavelength of 260 nm) structures of CHA as functions of the SDS concentration.

**Figure 3:**
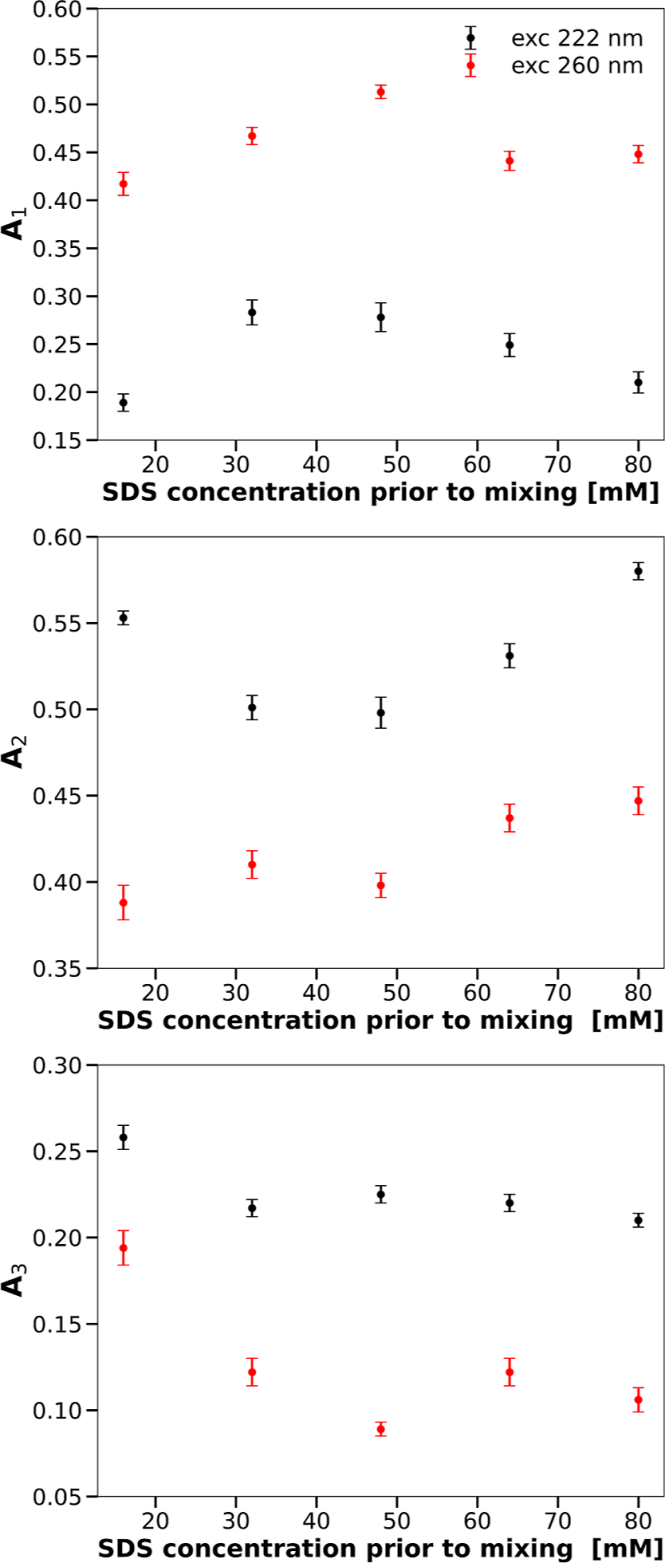
Relative relaxation amplitudes of the transitions in the secondary (black symbols, excitation wavelength of 222 nm) and the tertiary (red symbols, excitation wavelength of 260 nm) structures of CHA as functions of the SDS concentration.

Fitting of CD progress curves, obtained for the two excitation wavelengths, with exponential decays, ∑_*i*=1_^3^*A*_*i*_ exp(−*k*_*i*_*t*), leads to three rates *k*_*i*_ and three relative amplitudes *A*_*i*_ shown in Figures 2 and 3. A comparison of rate constants and amplitudes from these figures allows us to conclude that the kinetics of changes in the secondary structure of chymotrypsin under the influence of SDS differ from the kinetics of changes in the tertiary structure, unlike previously described by Takeda.^9^ Differences in the kinetics of secondary and tertiary structure changes are also visible from the comparison of the simultaneously recorded CD signal and fluorescence signal at 222 nm excitation. Let us also note that, looking separately at the dependence of the kinetic parameters on the SDS concentration used in the mixing experiments, for recording the CD and fluorescence signals, we do not see anything in Figures 2, 3, 6, 7, S8 and S9 that would indicate their sensitivity to the change in the structure of micelles from spherical to cylindrical that occurs in the range of SDS concentrations between 8 and 40 mM after mixing.^49^

The two different procedures applied to analyze experimental progress curves, i.e., numerical integration of differential equations describing structural transitions and fitting of multiexponential functions, result in the same number of steps. Rate constants and their standard errors obtained from the integration of differential equations are the same as the rate constants and standard errors resulting from the fitting of exponential functions.

#### 3.2.2 Kinetics of Tertiary Structure Changes. Fluorescence Measurements at 222 and 260 nm Excitation Wavelengths

Usually, when fluorescence measurements are performed in protein studies, the excitation wavelength is set to 280 nm or more. In our kinetic studies, CHA fluorescence is excited using wavelengths of either 222 or 260 nm because the fluorescence is recorded simultaneously with the CD signal. Due to the use of a 320 nm cutoff filter, we predominantly record the fluorescence of CHA tryptophans.^50^

Figure S5 shows the relative fluorescence reaction progress curves recorded after mixing a 40 μM chymotrypsin solution with SDS solutions of different concentrations for both excitation wavelengths. There is a clear drop in the registered signal occurring within the dead time of the stopped-flow spectrometer (the relative fluorescence progress curves do not start at zero), resulting from the influence of SDS molecules on the fluorescence of tryptophan chromophores.

High concentrations of CHA used in our kinetic experiments to obtain a good signal-to-noise ratio in the reaction progress curves recorded in CD measurements lead to the inner filter effect.^48^ This effect does not affect the reaction progress curves recorded in the fluorescence measurements. Due to the fact that CHA does not absorb radiation with wavelengths above 320 nm, the correction for the intensity of the measured fluorescence is limited to a constant factor, which remains the same for all wavelengths of the recorded fluorescence (eq 4).

However, fluorescence progress curves are disturbed by photobleaching,^51^ particularly at the excitation wavelength of 222 nm. The influence of photobleaching on the recorded progress curves is documented in Figure S6. We performed several 1000 s long observations of fluorescence after mixing a 10 μM CHA solution with either the phosphate buffer or with the 40 mM solution of SDS in the phosphate buffer using both the Chirascan spectrometer and the SX20 spectrometer. The SX20 spectrometer is equipped with a lamp which, at 222 nm wavelength, emits low-intensity light, whereas Chirascan’s light intensity at this wavelength is much higher. However, at 260 nm, the intensity of light emitted by both lamps is comparable. Figure S6 shows that the progress curves registered using the SX20 spectrometer at the 222 nm excitation wavelength reach an almost perfect plateau. There appears, however, to be no plateau in the progress curves recorded at this excitation wavelength with the Chirascan spectrometer. When the 260 nm excitation wavelength is used, the traces recorded with both spectrometers are quite similar and lack plateaus.

As the kinetics of photobleaching in a pure buffer and in the presence of SDS is different, effects of photobleaching cannot be subtracted from curves registered in kinetic experiments. This is clearly evidenced in the previously mentioned Figure S5. None of the relative fluorescence decays, obtained for indicated concentrations of CHA and SDS that are shown in the Graphs, reach a plateau.

The relative progress curves shown in Figure S6 were analyzed using the DynaFit program,^36,37^ either by fitting sums of exponential functions or in terms of solving systems of differential equations describing sequences of irreversible unimolecular transformations. Both approaches gave the same results. The discrimination procedure implemented in DynaFit gave a sequence of four unidirectional unimolecular transitions (or equivalently, an exponential decay with four relaxation times) as the most probable model of experimental observations. An exemplary fit of the best model and, for comparison, a monoexponential function are presented in Figure S7. The four rate constants and four relative relaxation amplitudes, which are functions of the SDS concentration and the excitation wavelength, are shown in Figures S8 and S9. From these figures, it is apparent that the obtained rates and amplitudes depend on the excitation wavelength.

The question therefore arises whether observed differences in the kinetics are solely the result of the photobleaching process, which for different excitation wavelengths occurs with different efficiencies, or whether there is another underlying molecular cause. We propose that the contribution of tryptophans differently located in the protein molecule to the total fluorescence of the molecule varies with the excitation wavelength. To test this hypothesis, we measured the steady-state fluorescence spectra of CHA, both in the pure buffer and in the presence of SDS, with the excitation wavelength set either to 222 or 260 nm. Next, the spectra were normalized and compared. Our reasoning is that if the various Trp residues are excited differently at these wavelengths, the equilibrium emission spectra of CHA should also be different.

**Figure 4:**
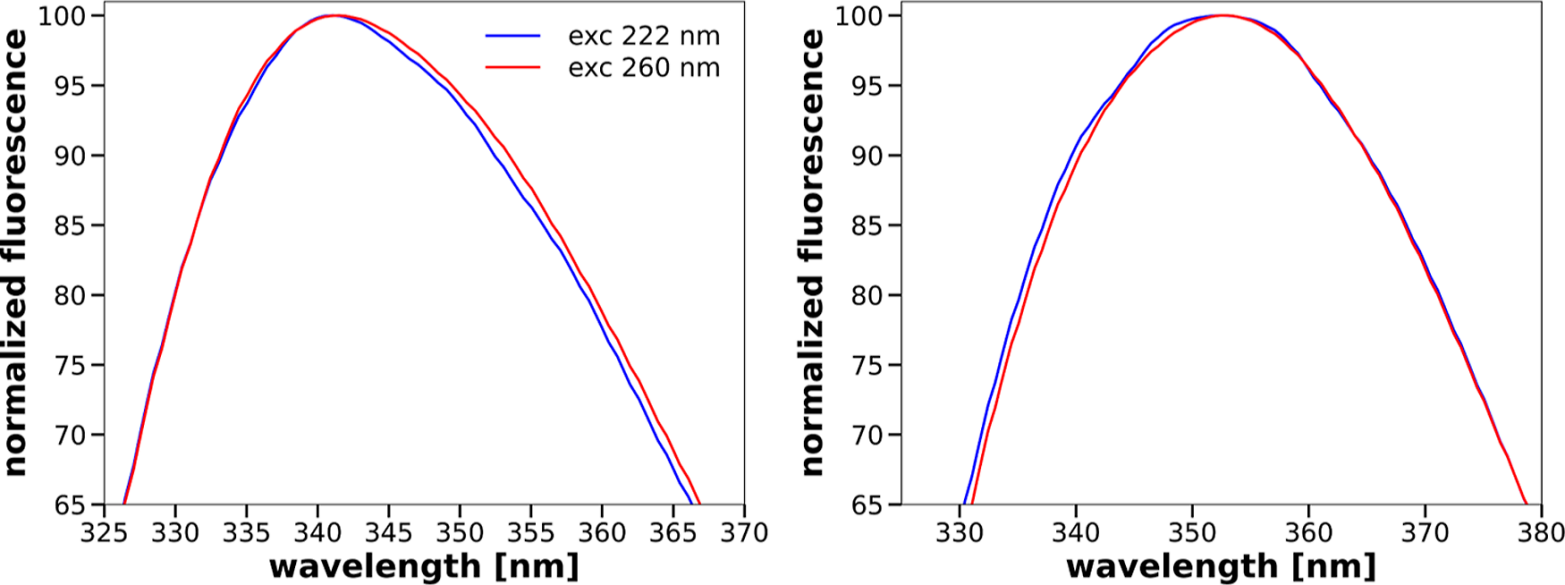
Normalized fluorescence equilibrium spectra of a 5 μM α-chymotrypsin solution at excitation wavelengths of 222 and 260 nm in phosphate buffer without (left) and with 20 mM SDS (right).

**Figure 5:**
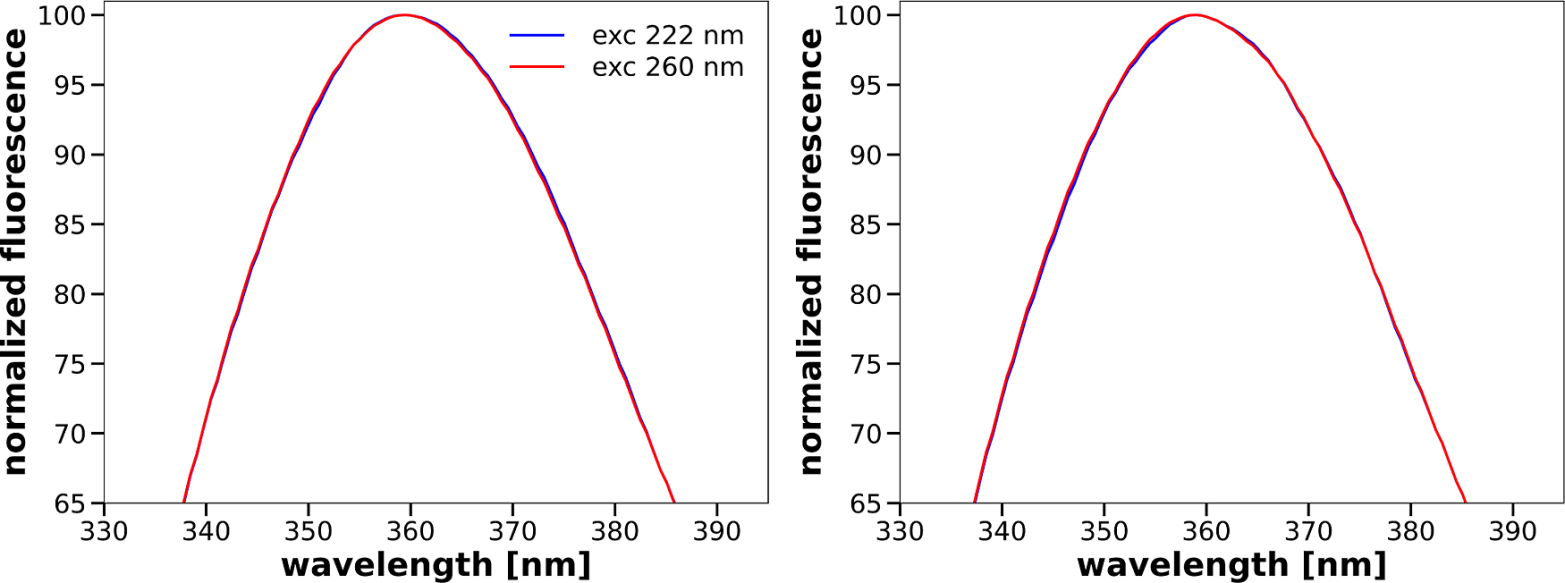
Comparison of normalized fluorescence spectra of 40 μM solutions of tryptophan in the phosphate buffer without (left) and with 20 mM SDS (right) for 222 and 260 nm excitation wavelengths.

Figure 4 gives such a comparison for a 5 μM solution of α-chymotrypsin in phosphate buffer without the addition of SDS and with an addition of 20 mM SDS. In both cases, the spectra depend on the excitation wavelength. However, differences between the spectra obtained for a given solution at the two excitation wavelengths are rather small (spectra are shown in a limited wavelength range around the fluorescence maximum). One of the possibilities to assess the significance of these differences would be to perform an analogous comparison of the pure tryptophan spectra. And to make this comparison more meaningful, we decided to use tryptophan solutions with the same concentration as the effective concentration of tryptophans in the considered CHA solution. Since CHA contains 8 tryptophans, its 5 μM concentration corresponds to a 40 μM tryptophan solution. The resulting tryptophan spectra are presented in Figure 5. In contrast to what can be seen in Figure 4, the normalized fluorescence spectra of 40 μM tryptophan solution in phosphate buffer without the addition of SDS and in phosphate buffer with the addition of 20 mM SDS do not depend on the excitation wavelength.

Figure S10 shows analogous data as Figure 4 for the CHA concentration of 20 μM and the SDS concentration of 40 mM. Again, the normalized fluorescence spectra of chymotrypsin in the phosphate buffer and in the phosphate buffer with the addition of SDS are different for excitation wavelengths of 222 and 260 nm. The equivalent tryptophan concentration for the 20 μM chymotrypsin solution is 160 μM. Such a high concentration would result in deviations in the absorption from the Lambert–Beer law. However, Figure S11 shows that this law is satisfied for 40 μM tryptophan solutions.

While the observations described above are in line with our hypothesis concerning the excitation wavelength-dependent contribution of different CHA tryptophans to the total fluorescence signal of the protein, it should be emphasized that further research is needed for its full validation.

#### 3.2.3 Kinetics of Tertiary Structure Changes. Comparison of CD and Fluorescence Measurements at the 260 nm Excitation Wavelength

Fluorescence reaction progress curves obtained after excitation at 260 nm were analyzed using the DynaFit program by fitting sums of up to three exponential functions, either with or without a linear term corresponding to photobleaching. Such an approach was taken by Otzen et al.^23^ for β-sheet proteins. These authors fitted the kinetics of SDS-induced structural transitions to monoexponential functions with a linear drift to account for the photobleaching of Trp fluorophores. A similar approach was taken by Patel and colleagues, who analyzed the ATP turnover cycle of kinesins.^40^ However, more complex models of photobleaching time dependence can be considered.^52−54^

As we have already described, the best model for the CD reaction progress curves consists of three irreversible unimolecular reactions. In the case of fluorescence measurements, the best model indicated by DynaFit is the following
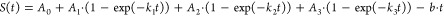


**Figure 6:**
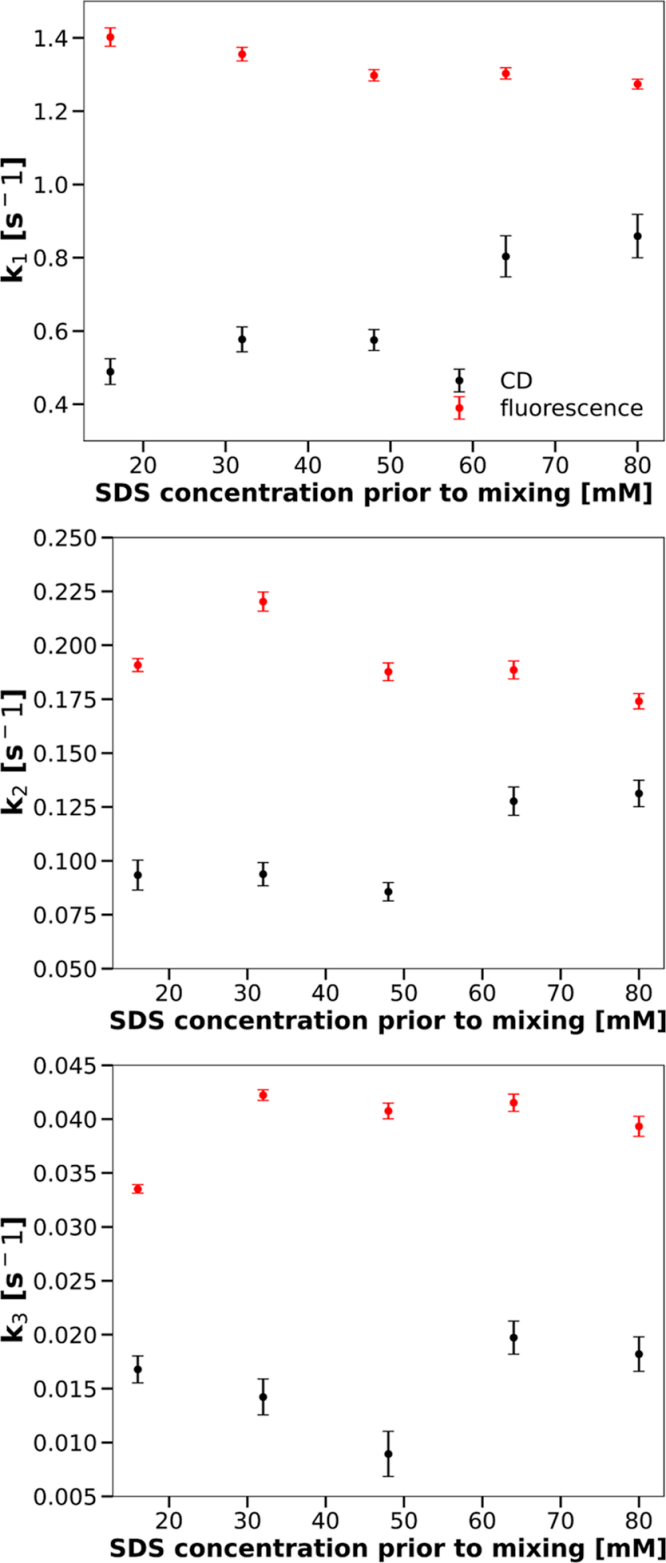
Rate constants for the CHA tertiary structure change as functions of SDS.

**Figure 7:**
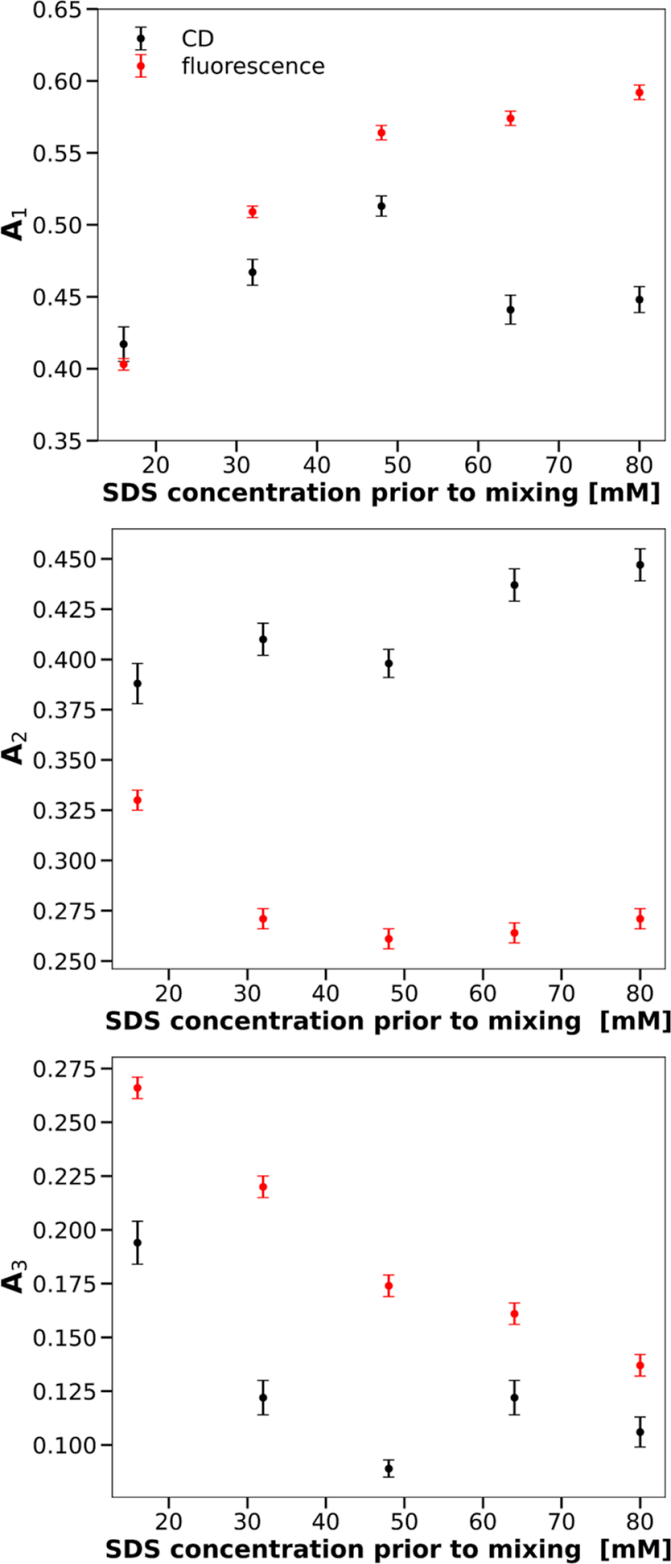
Amplitudes for the CHA tertiary structure change as a function of SDS.

Figures 6 and 7 present comparisons of the three rate constants and the three relative amplitudes characterizing SDS-induced changes in the CHA tertiary structure, resulting from an analysis of progress curves obtained by means of either CD or fluorescence measurements. The value of the *b* parameter decreases slightly with the increasing concentration of SDS—from 0.000369 ± 0.000005 [s^–1^] for 16 mM SDS in the solution prior to mixing to 0.000302 ± 0.000004 [s^–1^] for 80 mM SDS in the solution prior to mixing.

It is apparent from these two figures that the kinetics of transitions in the CHA tertiary structure resulting from the two types of measurements are different.

### 3.3 Experiments Focused on the Detection of the Free Protein Fraction in CHA–SDS Solutions

We performed kinetic mixing experiments designed to detect (if present) apoprotein molecules in CHA–SDS solutions. While Takeda and Moriyama^9^ stated that there is no free protein in solutions in which the SDS concentration is insufficient to saturate all binding sites of all proteins, Li and Lee^3^ allow for the presence of free protein molecules in protein–surfactant solutions.

**Figure 8:**
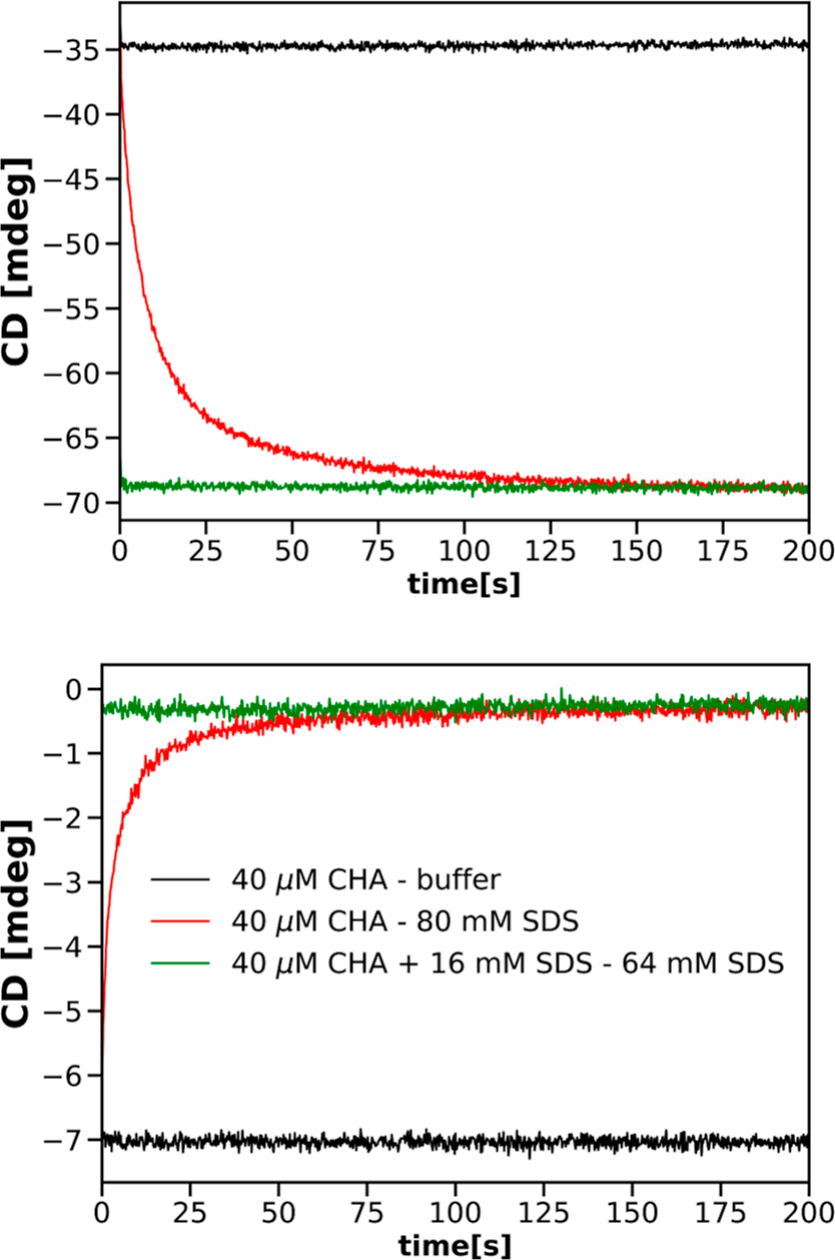
Progress curves obtained after mixing 40 μM CHA + 16 mM SDS with 64 mM SDS compared with progress curves obtained after mixing protein solutions with buffer and with 80 mM SDS. Top: excitation wavelength of 222 nm. Bottom: excitation wavelength of 260 nm.

In the first type of experiment, we mixed a buffer solution containing 40 μM CHA and 16 mM SDS with a solution of 64 mM SDS to obtain a buffer solution of 20 μM CHA and 40 mM SDS. Figure 8 shows the CD progress curves observed in these experiments for excitation wavelengths of 222 and 260 nm. None of these curves exhibit relaxation behavior. These curves are compared with two other progress curves (Figure 8): one recorded after rapid dilution of the 40 μM CHA solution with the buffer, and the second one recorded after mixing buffer solutions of 40 μM CHA and 80 mM SDS.

If there were proteins with no bound SDS molecules in a given CHA-SDS mixture, as claimed by some authors,^3^ we could expect that the fraction of the apoprotein at 16 mM SDS (prior to the mixing) would be greater than the fraction at 40 mM SDS (after the mixing). Also, we were able to notice a relaxation in mixing experiments involving 64 mM SDS solutions. Following Otzen,^18,19,23^ let us consider the binding step of the SDS-CHA interaction
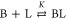
where
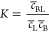
with B denoting CHA, L denoting SDS, and  being the equilibrium concentration of species X. From the mass conservation principle, we have for initial concentrations:  and , hence
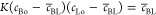
5

Let us now assume that for one particular concentration, *c*_Lo_, the resulting equilibrium concentration, , is *xc*_Bo_, where *x* is between 0 and 1. Equation 5 can be written as
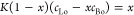


Now, we increase the total concentration of ligand *n* times. What is the fraction of bound protein *x*_*n*_ for this increased concentration of the ligand? We have
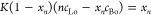
and as *x* is between 0 and 1, *c*_Bo_ is in the micromolar range, and *c*_Lo_ is in the millimolar range, we can neglect *xc*_Bo_ in comparison with *c*_Lo_ and *x*_*n*_*c*_Bo_ in comparison to *nc*_Lo_. This leads to
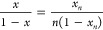
and so
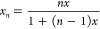


**Figure 9:**
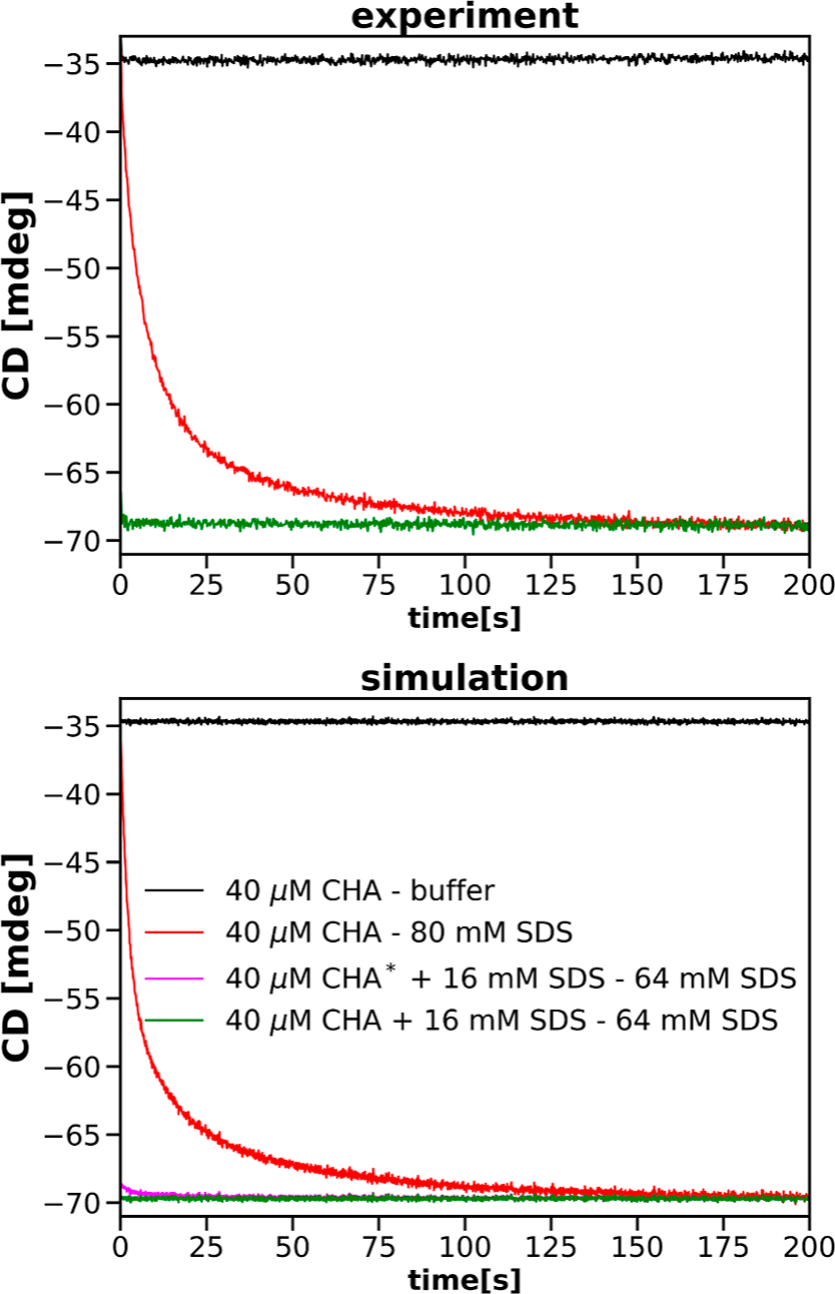
Comparison of experimental (green) and simulated (green and magenta) CD progress curves for mixing 40 μM CHA + 16 mM SDS solution with 64 mM solution of SDS. The magenta progress curve was simulated assuming that 1.2 μM CHA of 40 μM in the solution with 16 mM SDS is in the apo form. In the figure legend, this simulation is additionally marked with an asterisk. The simulated green progress curve is obtained assuming there is no apoprotein in the solution. The experimental and simulated progress curves for mixing 40 μM CHA solution with the buffer (black) or 80 mM SDS solution (red) are included for reference. The excitation wavelength is 222 nm.

Let *x* = 0.9 for 16 mM SDS. Increasing the SDS concentration 2.5 times (to 40 mM), as in our experiment, we should obtain *x*_*n*_ = 0.9574, i.e., the concentration of CHA bound to SDS is increased by almost 6%. For a comparison, if *x* = 0.95, then *x*_*n*_ = 0.9794, i.e., the concentration of CHA bound to SDS is increased by almost 3%. We simulated using DynaFit what progress curves would expected to be recorded after mixing the 40 μM CHA + 16 mM SDS solution with the 64 mM. Figure 9 shows a comparison between the experimental progress curves (presented already in the upper part of Figure 8) and the corresponding simulated progress curves. Please note the green and magenta progress curves shown in the bottom part of Figure 9. The green curve results from simulations of mixing the 40 μM CHA and 16 mM SDS buffer solution with the 64 mM SDS solution with no free protein in the former solution. This corresponds to the green curve in the top part of Figure 9. The magenta progress curve results from a similar mixing, but with the assumption that 5% of CHA in the first solution (i.e., 2 μM) is in the apo state. After the mixing, the 0.6 μM fraction of the apoprotein binds SDS molecules. The green and magenta progress curves differ in their initial course.

A detailed comparison of the progress curve simulated for the nonzero apoprotein fraction with the curve obtained experimentally is shown in Figure S12. It appears that the 5% population of free CHA in the solution of 20 μM CHA with 8 mM SDS at time 0 in the stopped-flow mixing cell should be detected in our experiments. While we cannot exclude that apo-CHA is also present in a solution of 40 μM CHA + 16 mM SDS, its fraction (if any) is too low to be detected by the above approach. Simultaneously, 16 mM SDS is not the saturating concentration for 40 μM CHA, as mixing the 40 μM CHA + 16 mM SDS solution with the 64 μM SDS solution results in a substantial change in the CD signal. However, this change does not have the form of relaxation curve. We conclude that our results are consistent with the statement of Takeda and Moriyama that in mixtures of micromolar solutions of proteins with millimolar concentrations of SDS, there is no free protein present.^9^

In the experiment described above, we mixed the 40 μM CHA solution containing the addition of *x* mM SDS with the solution of 80 – *x* mM SDS, with *x* = 16 mM. We did not observe relaxation. We also performed experiments with *x* sufficiently small so that after mixing the 40 μM CHA solution containing the addition of *x* mM SDS with the solution of 80 – *x* mM SDS, we saw the relaxation behavior. Our aim was to determine the threshold x value above which relaxation cannot be observed. One should note that in all such experiments, the final concentrations of CHA and SDS are 20 μM and 40 mM, respectively. We made these experiments only with the 222 nm excitation wavelength because of the better signal-to-noise ratio than that obtained for the 260 nm excitation wavelength. The results are discussed in the following paragraphs.

**Figure 10:**
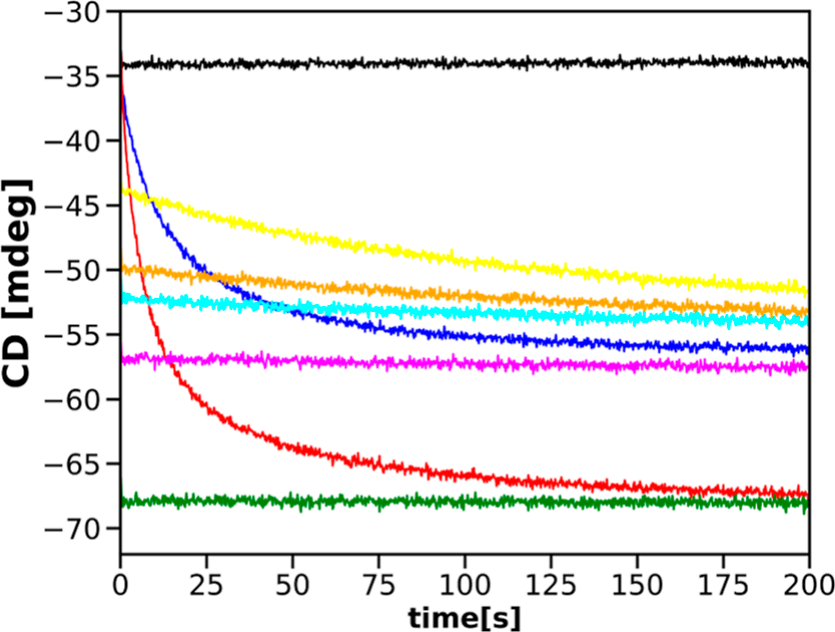
Progress curves recorded for a set of mixing experiments leading to the final 40 mM concentration of SDS, together with reference progress curves: 40 μM CHA solution mixed with the buffer, 40 μM CHA solution mixed with the 80 mM SDS solution, 40 μM SDS solution mixed with the 8 mM SDS solution, 40 μM CHA + 8 mM SDS solution mixed with the 72 mM SDS solution, 40 μM CHA + 16 mM SDS solution mixed with the 64 mM SDS solution, 40 μM CHA + 6 mM SDS solution mixed with the 74 mM SDS solution, 40 μM CHA + 5 mM SDS solution mixed with the 75 mM SDS solution, and 40 μM CHA + 4 mM SDS solution mixed with the 76 mM SDS solution.

Figure 10 shows stopped-flow progress curves recorded at 222 nm after mixing the 40 μM CHA + *x* mM SDS solution and a (80 – *x*) mM SDS solution, where *x* = 0, 4, 5, 6, 8, and 16 mM. Additionally, we show progress curves obtained after mixing the 40 μM CHA solution with the buffer and with the 8 mM SDS solution.

As can be seen, progress curves recorded after mixing 40 μM CHA with the addition of *x* mM SDS and (80 – *x*) mM SDS for *x* = 4, 5, and 6 mM exhibit relaxation behavior. However, their tail values are well above the plateau observed for mixing the 40 μM CHA solution with the 80 mM solution of SDS, even though the final concentrations of SDS in these two cases are the same. Progress curves recorded after mixing the 40 μM CHA + *x* mM SDS solution with the (80 – *x*) mM SDS solution for *x* = 8 and 16 mM exhibit no relaxation. The signal recorded after mixing the 40 μM CHA + 8 mM SDS solution with the 72 mM SDS solutions is close to the plateau value of the progress curve obtained for mixing the 40 μM CHA solution with the 8 mM SDS solution. The signal recorded after mixing the 40 μM CHA + 16 mM SDS solution and the 64 mM SDS solution is close to the plateau value of the progress curve obtained after mixing the 40 μM CHA solution with the 80 mM SDS solution, as already shown in Figure 8.

**Figure 11:**
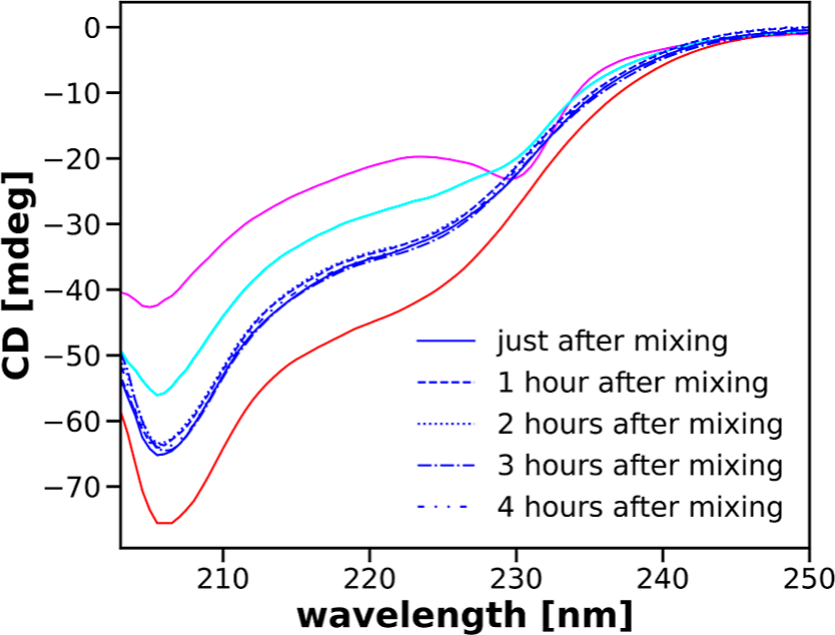
CD spectra in the far ultraviolet region obtained after mixing the 40 μM CHA solution with the buffer, 40 μM CHA solution with the 80 mM solution of SDS, 40 μM CHA + 4 mM SDS solution with the buffer, and 40 μM CHA + 4 mM SDS solution with the 76 mM solution of SDS. The last spectrum was recorded 5 times in 1 h intervals, the first time just after the mixing.

The results shown in Figure 10 indicate that the final protein state obtained after mixing with SDS depends not only on the final concentration of the surfactant but also on the mixing procedure. These different final states seem relatively stable, as confirmed by Figure 11 and our previous investigations.^55^

Figure 11 shows the stationary CD spectra obtained after mixing the 40 μM CHA + 4 mM SDS solution with the solution of 76 mM SDS. These spectra are stable for at least 4 h after the mixing. Moreover, they are different from the spectrum obtained after mixing the 40 μM CHA solution with the 80 mM solution of SDS, even though the final concentration of SDS in both cases is 40 mM. For a comparison, we show spectra recorded after mixing the 40 μM CHA solution with the buffer and after mixing the 40 μM CHA + 4 mM SDS solution with the buffer. The four spectra are different, indicating differences in the secondary structure of α-chymotrypsin. The most important is the difference between the protein structure obtained after mixing the 40 μM CHA + 4 mM SDS solution with the solution of 76 mM SDS and the structure obtained after mixing the 40 μM CHA solution with the 80 mM SDS solution. These spectra are stable for at least several hours.

## 4 Conclusions

The key findings of our work can be summarized as follows.

Apparently, SDS-induced transitions in the secondary and tertiary structures of CHA, investigated by means of CD in the far- and near-UV, occur with different rate constants, unlike those described previously by Takeda.^11^

The kinetics of transitions in the tertiary structure of CHA, resulting from fluorescence measurements, depend on the excitation wavelength, which can only partially be explained as a consequence of photobleaching. We hypothesize that the contributions of the protein’s different tryptophans to the total recorded fluorescence depend on the excitation wavelength. This opens a field for novel studies that can either further validate or negate our explanation. The validity of our hypothesis would enable the creation of measurement protocols, allowing detailed observations and analysis of structural transitions in proteins.

Fluorescence measurements and CD measurements give different kinetics of tertiary structure transitions for an excitation wavelength of 260 nm.

We confirm the previous conclusion of Takeda^9^ that there is a threshold surfactant concentration beyond which there are no free proteins in the protein–SDS solution.

On a final note, we hope that the findings described in the current work will inspire further studies on protein–surfactant interactions. In particular, it would be interesting to focus on the influence of pH and ionic strength on the kinetics of surfactant-induced transitions in the secondary and tertiary structures of proteins.

## Supporting Information Available

Stationary CD, fluorescence, and absorption spectra of α-chymotrypsin or tryptophan solutions with or without the addition of SDS, CD or fluorescence recorded progress curves, fitting of exemplary fluorescence progress curves with single and sum of four exponential functions, the dependence of kinetic parameters (rate constants and relaxation amplitudes) derived from fitting fluorescence progress curves with the sum of four exponential functions, and simulated and experimental CD progress curves supporting the conclusion that there are no free chymotrypsin molecules above a certain threshold while simultaneously not saturating the concentration of SDS (PDF)
