# Supplementary material for: Kinetics of Structural Transitions Induced by Sodium Dodecyl Sulfate in α-Chymotrypsin
Source: ACS Omega. 2023 Dec 13;8(51):49137–49. doi: 10.1021/acsomega.3c07256 (PMC10753550; doi:10.1021/acsomega.3c07256)
Supplement: Supplementary file 1 — ao3c07256_si_001.pdf [file ao3c07256_si_001.pdf]

Supporting information for: Kinetics of Structural Transitions Induced by Sodium Dodecyl Sulfate in α-Chymotrypsin.

Karolina Stachurska, Urszula Marcisz, Maciej Długosz, and Jan M. Antosiewicz*

Biophysics Division, Institute of Experimental Physics, Faculty of Physics, University of Warsaw, Pasteura 5 St., 02-093 Warsaw, Poland

E-mail: jantosi@fuw.edu.pl

*Corresponding author: Jan M. Antosiewicz

This file includes Supporting Figures S1:S12

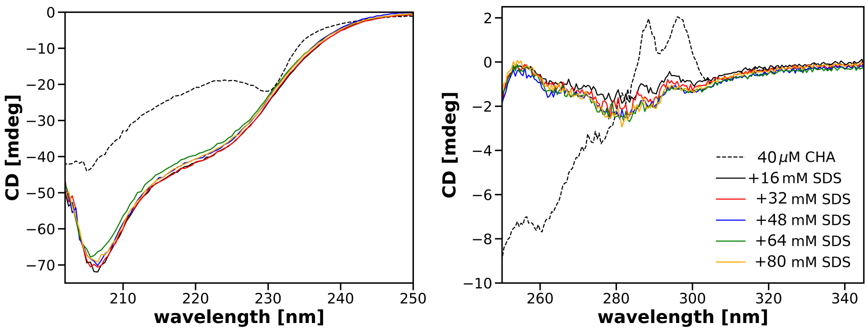

Figure S1: Circular dichroism spectra in the far- and near-ultraviolet region obtained for 20 μM solution of α-chymotrypsin (CHA) in phosphate buffer, for different concentrations of SDS.

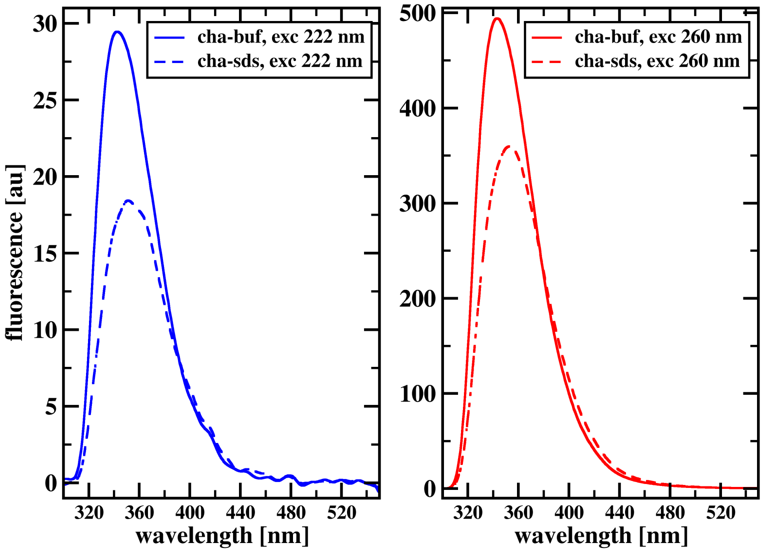

Figure S2: Fluorescence spectra for two excitation wavelengths, 222 and 260 nm, obtained for 20 μM solution of α-chymotrypsin (CHA) in phosphate buffer, and in phosphate buffer with 40 mM SDS, recorded using a Schott WG 320 nm cut-off filter.

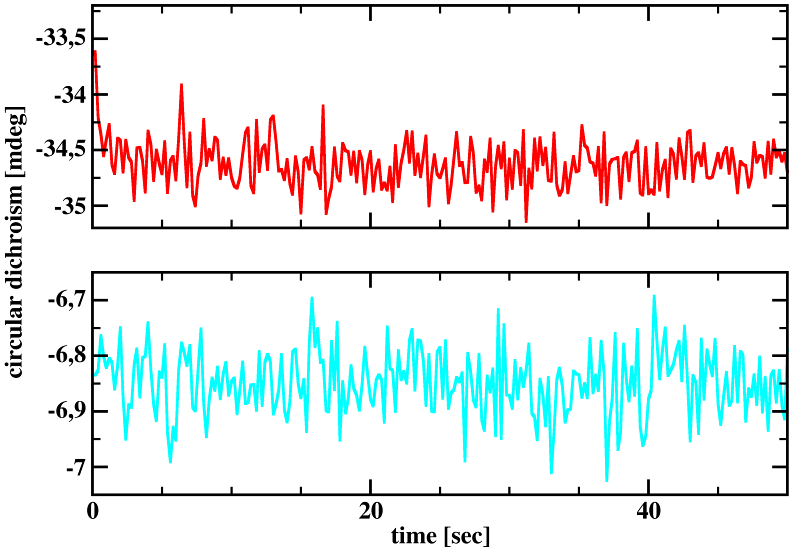

Figure S3: The first 50 seconds of circular dichroism progress curves (upper row 222 nm; bottom row 260 nm) obtained after mixing the 40 μM solution of α-chymotrypsin with phosphate buffer.

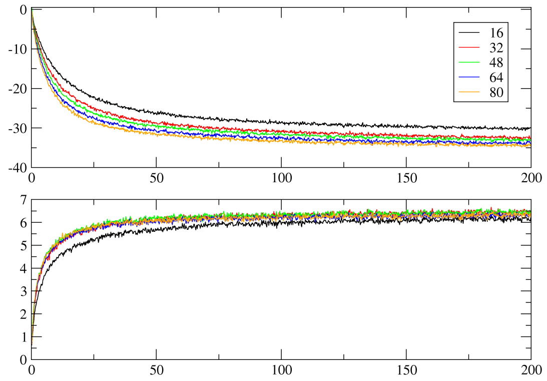

Figure S4: Relative circular dichroism progress curves (upper row 222 nm; bottom row 260 nm) obtained after mixing the 40 μM solution of α-chymotrypsin with solutions of SDS (SDS concentrations in the 16–80 mM range). Legend shows concentrations of SDS in mM, just prior to mixing.

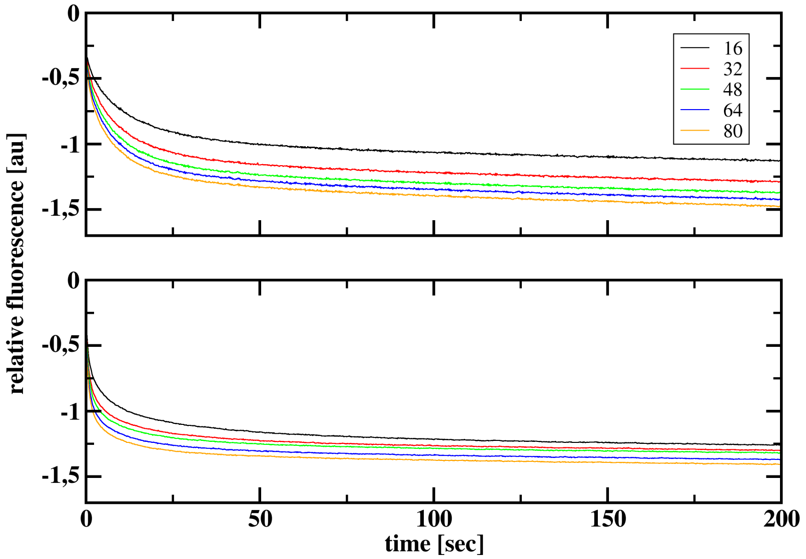

Figure S5: Relative fluorescence progress curves (upper row exc 222 nm; bottom row exc 260 nm) obtained after mixing the 40 μM solution of α-chymotrypsin with solutions of SDS (SDS concentrations in the 16–80 mM range). Legend shows concentrations of SDS in mM, just prior to mixing.

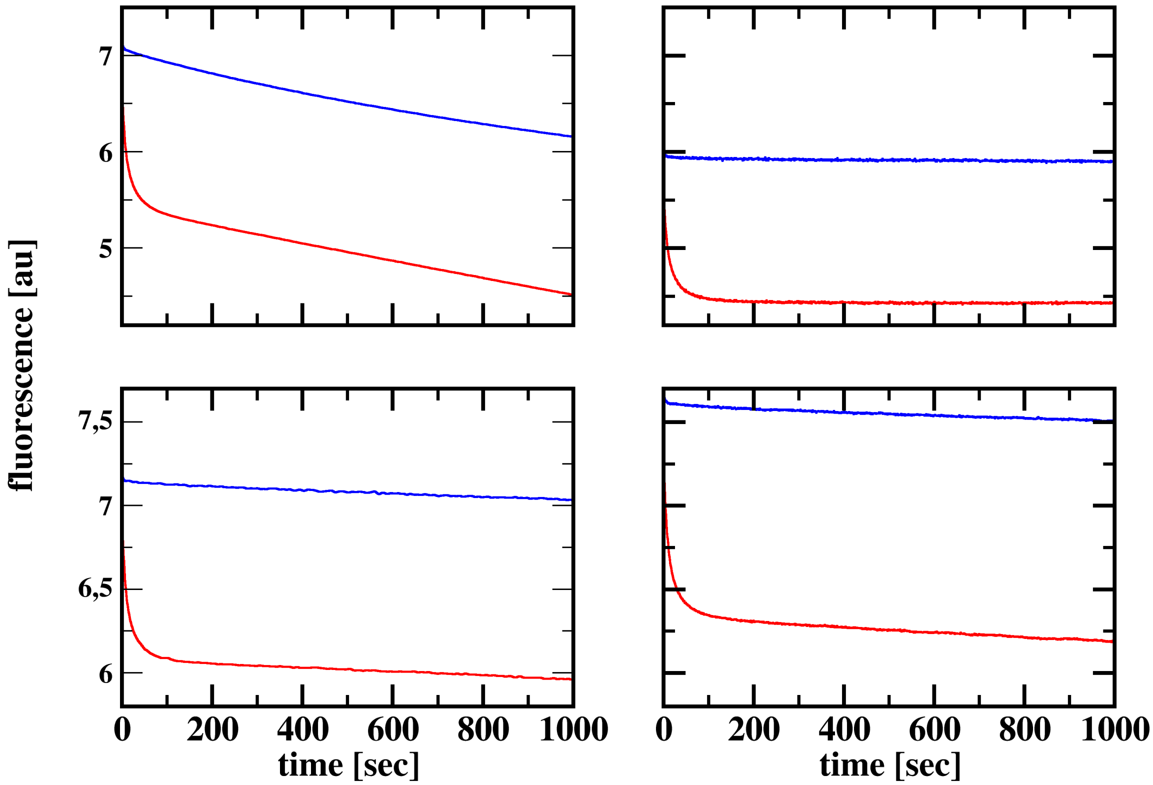

Figure S6: Bufor corrected fluorescence progress curves recorded by Chirascan spectrometer (left column) and SX20 spectrometer (right column), with excitation 222 nm (upper row) and 260 nm (bottom row), after mixing 10 μM α-chymotrypsin with phosphate buffer (blue) and 10 μM α-chymotrypsin with 40 mM SDS in phosphate buffer (red).

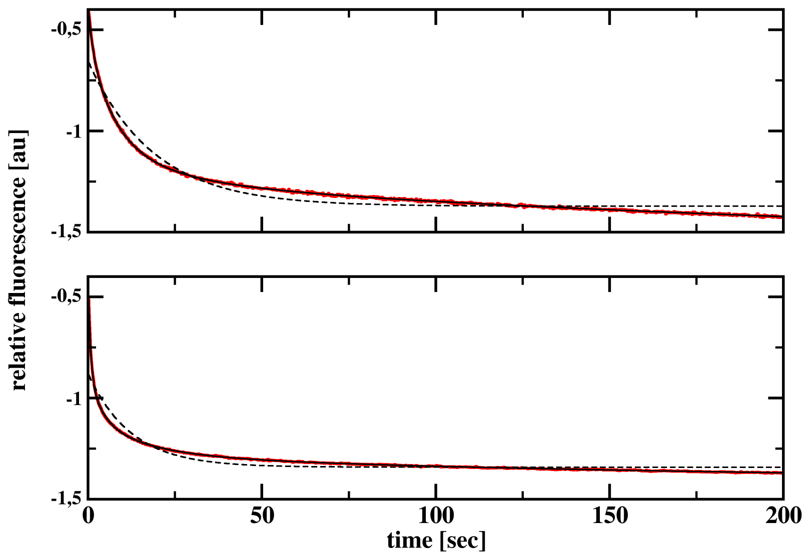

Figure S7: Examplary DynaFit fits of relative fluorescence progress curves (222 nm upper and 260 nm lower) corresponding to irreversible four-step transitions, to the the progress curves obtained after mixing the 40 μM solution of α-chymotrypsin with 64 mM solution of SDS. Best fits corresponding to a one-step transition are shown for a comparison.

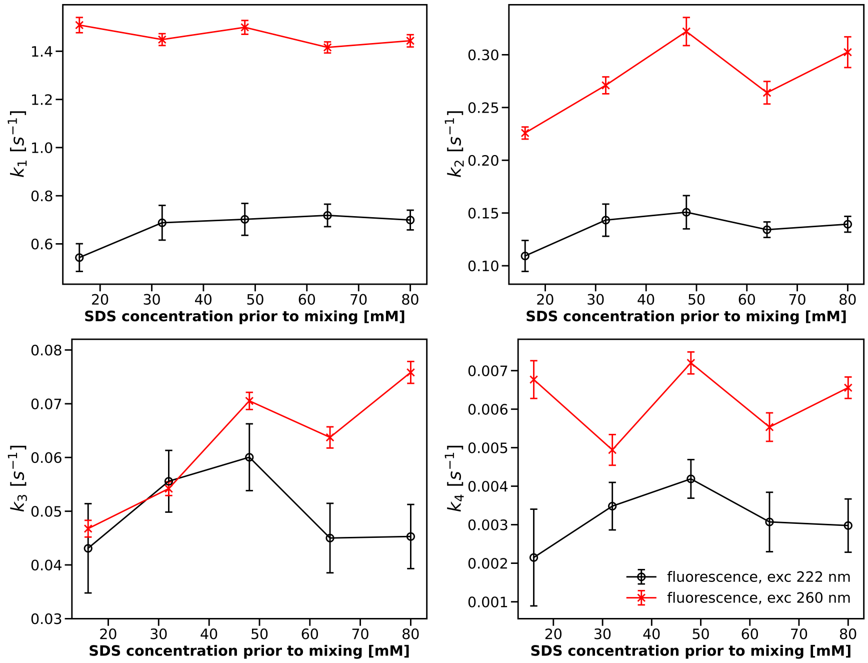

Figure S8: Dependence of the four transition rate constants derived from fitting fluorescence progress curves with the DynaFit program on the SDS concentration. Blue lines: fluorescence, 222 nm excitation wavelength. Red lines: fluorescence, 260 nm excitation wavelength.

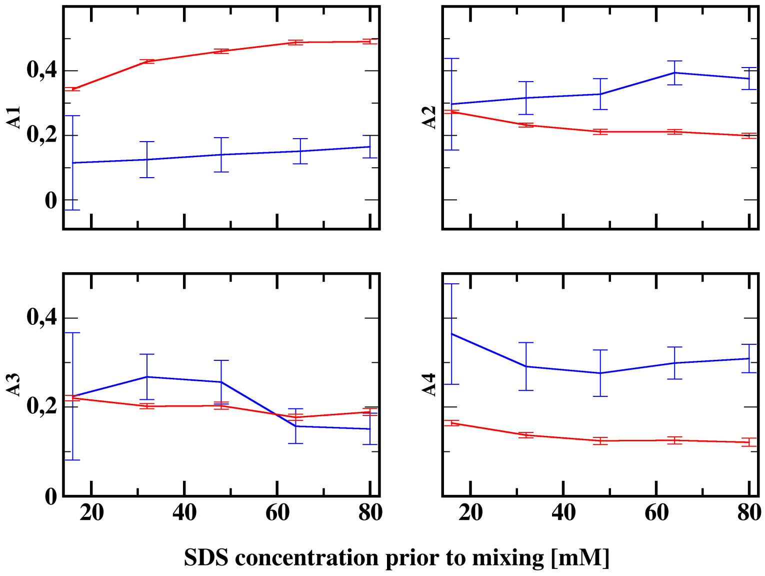

Figure S9: Dependence of the four relaxation amplitudes, derived from fitting sum of four exponents to the fluorescence progress curves with the DynaFit program, on the SDS concentration.

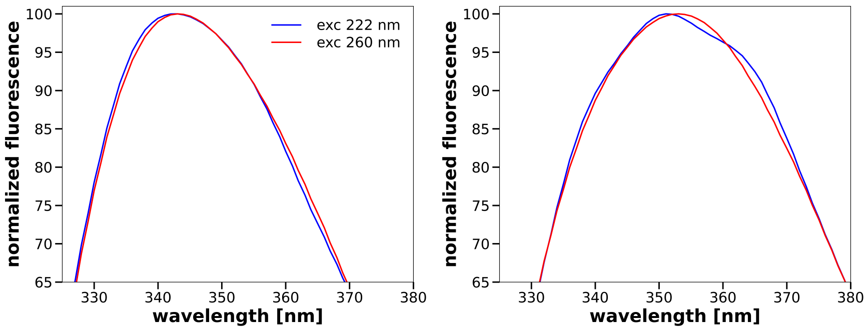

Figure S10: Normalized fluorescence equilibrium spectra of 20 μM α-chymotrypsin solution with excitation vavelength of 222 and 260 nm in 10 mM phosphate buffer without (left) and with 40 mM SDS (right).

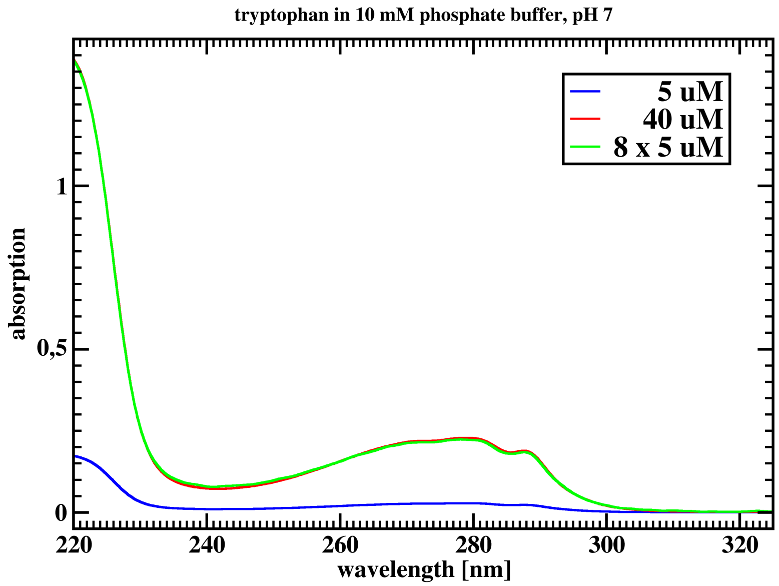

Figure S11: Comparison of the absorption spectrum of a 40 μM solution of tryptophan in phosphate buffer with the spectrum at a concentration of 5 μM, multiplied by 8.

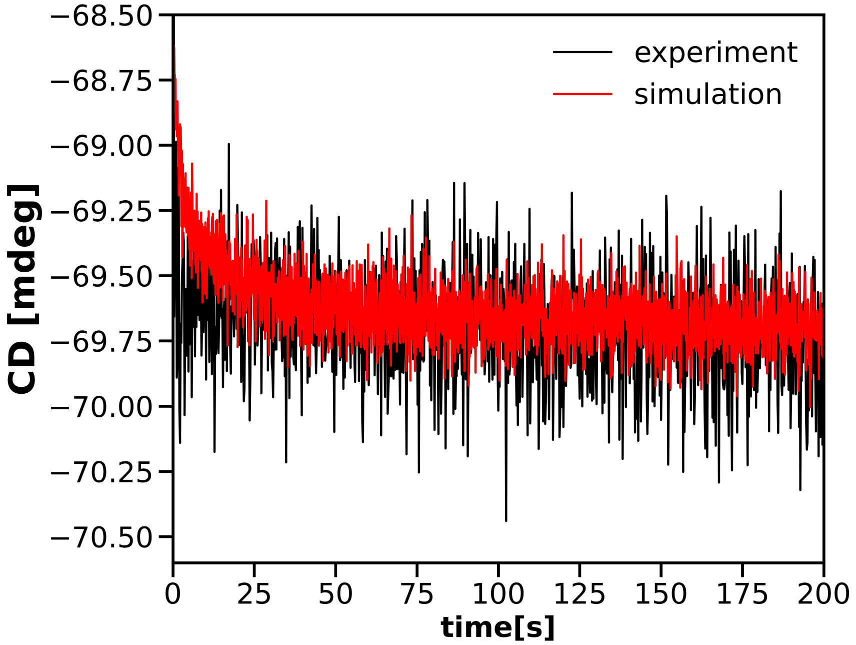

Figure S12: Details of the experimental and simulated progress curves for mixing the 40 μM CHA + 16mM SDS solution with the 64 mM SDS solution, for the apo-CHA concentration of 0.6 μM immediately after the mixing. Excitation wavelength 222 nm.
